# Da-Chai-Hu-Tang Protects From Acute Intrahepatic Cholestasis by Inhibiting Hepatic Inflammation and Bile Accumulation *via* Activation of PPARα

**DOI:** 10.3389/fphar.2022.847483

**Published:** 2022-03-15

**Authors:** Shihao Xu, Xi Qiao, Peike Peng, Ziyi Zhu, Yaoting Li, Mengyuan Yu, Long Chen, Yin Cai, Jin Xu, Xinwei Shi, Christopher G. Proud, Jianling Xie, Kaikai Shen

**Affiliations:** ^1^ School of Basic Medical Sciences, Shanghai University of Traditional Chinese Medicine, Shanghai, China; ^2^ School of Pharmacy, Fudan University, Shanghai, China; ^3^ School of Biosciences, University of Birmingham, Birmingham, United Kingdom; ^4^ Experimental Center for Science and Technology, Shanghai University of Traditional Chinese Medicine, Shanghai, China; ^5^ Department of Health Technology and Informatics, The Hong Kong Polytechnic University, Kowloon, Hong Kong SAR, China; ^6^ Lifelong Health Theme, South Australian Health and Medical Research Institute, Adelaide, SA, Australia; ^7^ Molecular and Biomedical Sciences, School of Biological Sciences, University of Adelaide, Adelaide, SA, Australia; ^8^ Flinders Health and Medical Research Institute, Flinders University, Adelaide, SA, Australia

**Keywords:** Da-Chai-Hu-Tang, intrahepatic cholestasis, liver injury, bile acid homeostasis, peroxisome proliferator-activated receptor alpha

## Abstract

Cholestasis is caused by intrahepatic retention of excessive toxic bile acids and ultimately results in hepatic failure. Da-Chai-Hu-Tang (DCHT) has been used in China to treat liver and gallbladder diseases for over 1800 years. Here, we demonstrated that DCHT treatment prevented acute intrahepatic cholestasis with liver injury in response to α-naphthylisothiocyanate (ANIT) not to bile duct ligation (BDL) induced-extrahepatic cholestasis. ANIT (80 mg/kg) increased serum levels of alanine aminotransferase (ALT), aspartate aminotransferase (AST), direct bilirubin (DBiL), total bilirubin (TBiL), and total bile acids (TBA) which was attenuated by DCHT treatment in a dose-dependent manner. DCHT treatment at high dose of 1.875 g/kg restored bile acid homeostasis, as evidenced by the recovery of the transcription of genes implicated in bile acid biosynthesis, uptake and efflux. DCHT treatment (1.875 g/kg) reversed ANIT-evoked disordered glutathione homeostasis (as determined by GSH/GSSG ratio) and increased in the mRNA levels for *Il6*, *Il1b* and *Tnfa* associated with liver inflammation. Using network pharmacology-based approaches, we identified 22 putative targets involved in DCHT treatment for intrahepatic cholestasis not extrahepatic cholestasis. In addition, as evidenced by dual-luciferase reporter assays, compounds from DCHT with high affinity of PPARα increased luciferase levels from a PPARα-driven reporter. PPARα agonist fenofibrate was able to mimic the cytoprotective effect of DCHT on intrahepatic cholestasis, which was abolished by the PPARα antagonist GW6471. KEGG enrichment and western blot analyses showed that signaling axes of JNK/IL-6/NF-κB/STAT3 related to PPARα might be the principal pathway DCHT affects intrahepatic cholestasis. Taken together, the present study provides compelling evidence that DCHT is a promising formula against acute intrahepatic cholestasis with hepatotoxicity which works *via* PPARα activation.

## Introduction

Cholestasis, which results from diminished bile formation from the hepatocytes, impairs bile secretion at the level of cholangiocytes and causes the obstruction of bile flow as a result of stone formation (cholelithiasis) or tumor bulk (Trauner et al., 1998). Cholestasis can be broadly divided into two subtypes, intrahepatic cholestasis and extrahepatic cholestasis. Intrahepatic cholestasis is a common feature in viral hepatitis, and drug or alcohol-induced liver disease, primary biliary cirrhosis (PBC), cholestasis during pregnancy and late stage of Hepatocellular Carcinoma (HCC) (European Association for the Study of the Liver, 2009; Liu et al., 2018). Extrahepatic cholestasis, characterized by dilated bile ducts, is caused by either a bile duct stones or stricture, with stricture most often related to a malignancy (Karvonen et al., 2006). This occurs when the balance of production and transport of bile acids is disrupted, leading to liver fibrosis, cirrhosis and liver failure (Li and Apte, 2015). Currently available therapeutic interventions (approved by the Food and Drug Administration (FDA)) against cholestasis include ursodeoxycholic acid (UDCA) and obeticholic acid (OCA), a farnesoid X nuclear receptor (FXR) agonist. UDCA slows down the progression of PBC, particularly during stage I and II of the disease. However, up to 40% of PBC patients remain irresponsive to UDCA (Ghonem et al., 2015; Wagner and Fickert, 2020). Hepatic transporters, including Na^+^-dependent taurocholate cotransporting polypeptide (NTCP), organic anion transporting polypeptide 2 (OATP2), bile acids export pump (BSEP) and multidrug resistance-associated protein 2 (MRP2), located along basolateral (sinusoidal) and apical (canalicular) membranes of hepatocytes, are integral determinants of bile formation and secretion (Nathanson and Boyer, 1991). Nuclear receptors (NRs) are critically involved in the regulation of the expression of these hepatic transporters and are targets for therapies against cholestatic liver diseases. One of these NRs is the peroxisome proliferator-activated receptor alpha (PPARα), which plays a pivotal role in maintaining the metabolic homeostasis of cholesterol, lipids, phospholipids and bile acids (Ghonem et al., 2015), by acting as a transcription factor that associates with the promoter of *Cyp7a1* (Cheema and Agellon, 2000), which encodes CYP7A1, a cytochrome P450 enzyme that controls cholesterol metabolism. The anti-inflammation response associated with activated PPARα is decreased in cholestasis (Ghonem et al., 2015). Indeed, PPARα agonist fenofibrate can be therapeutically beneficial for the treatment of various cholestatic liver disorders (Ghonem et al., 2015). On the other hand, STAT3 lies downstream of PPARα and can be activated by the release of inflammatory cytokines, such as IL-1ß, IL-6 and TNF-α (Yu et al., 2002).

Da-Chai-Hu-Tang (DCHT), a classic Traditional Chinese medicine (TCM) formula, has been applied for treating liver and gallbladder diseases for over 1,800 years in China. It is comprised of Bupleuri Radix (Chai Hu), Scutellariae Radix (Huang Qin), Paeoniae Radix Alba (Bai Shao), Pinelliae Rhizoma (Ban Xia), Rhei Radix et Rhizoma (Da Huang), Aurantii Fructus Immaturus (Zhi Shi), Zingiberis Rhizoma Recens (Sheng Jiang), and Jujubae Fructus (Da Zao) (Yoshie et al., 2004; He et al., 2014). DCHT is commercially available in both Japan and China. It exhibits multifaceted pharmacological bioactivities against diseases such as pancreatitis, hypercholesterolemia, diabetes, hyperlipidemia, gastritis, habitual constipation, obesity, etc. (Umeda et al., 1989; Yoshie et al., 2004; Duan et al., 2017; Han et al., 2020; Lian et al., 2020). Previous clinical study showed that DCHT improved clinical outcome against cholestatic liver injury (Song et al., 2019). The characteristic of formula DCHT is a combinational therapeutical strategy that comprise of more than one active ingredient to improve clinical efficacy; however, it has been challenging to establish the underlying mechanism(s) by which DCHT exerts its therapeutic effects.

Network pharmacology, a Rosetta stone for TCM formulas, integrates pharmacodynamics and pharmacokinetics to stands on a systematic and integrative viewpoint towards the intervention and the effects of TCM formulas on the treatment for complicated diseases (Hao and Xiao, 2014). Such a strategy resonates with the holistic view of TCM and the concept of “multi-compound, multi-pathway and multi-target synergy” in TCM (Li and Zhang, 2013). In this study, we applied a network pharmacology to characterize the protective properties of DCHT against acute intrahepatic cholestasis with liver injury, we also applied several biochemical approaches to investigate the underlying mechanisms.

## Materials and Methods

### Compounds, Reagents and Antibodies

Da-Chai-Hu-Tang (Dai-saiko-to in Japanese) (Lot: M25161) was purchased from Tsumura & Co. (Tokyo, Japan). The dried decoction of DCHT formula with eight traditional Chinese herbs contains 6 g Root of Bupleurum falcatum L [Umbelliferae; Bupleuri Radix], 4 g Tuber of Pinellia ternata (Thunb.) Makino [Araceae; Pinelliae Rhizoma], 3 g Root of Scutellaria baicalensis Georgi [Labiatae; Scutellariae Radix], 3 g Root of Paeonia lactiflora Pall [Paeoniaceae; Paeoniae Radix Alba], 3 g Fructus of Ziziphus jujuba Mill [Ramnaceae; Jujubae Fructus], 2 g Fructus Immaturus of Citrus × aurantium L [Rutaceae; Aurantii Fructus Immaturus], 1 g Rhizoma of Zingiber officinale Roscoe [Zingiberaceae; Zingiberis Rhizoma Recens], and 1 g Rhizoma of Rheum palmatum L [Polygonaceae; Rhei Radix et Rhizoma], which were added to 700 ml of water, boiled for 1 h, filtered and then concentrated to 300 ml. This decoction was spray-dried to yield 4.5 g of a powdered extract, which represents a 1-day dosage as previously described (He et al., 2014).

Narirutin, baicalein, rhein, wogonin, chrysophanol, naringenin, kaempferol, saikosaponin A, emodin-3-methyl ether, paeoniflorin and emodin were purchased from Shanghai R&D Centre for Standardization of Chinese Medicines (Shanghai, China). These structures were determined using ^1^H-NMR and ^13^C-NMR spectral analysis, and its purity was more than 98% as determined by high pressure liquid chromatography analysis. All compounds were dissolved in absolute dimethyl sulfoxide (DMSO) as 100 mM, and, on the experimental day, was further diluted with culture medium.

Please see Supplementary Table S1 for a complete list of reagents and antibodies used in this study.

### LC-MS Based Chemoprofile

DCHT (0.5 g) was extracted with 2 ml of methanol under ultrasonication for 30 min, and then centrifuged at 10,000 × *g*, at 4°C for 15 min. High-resolution liquid-chromatography-mass spectrometry (LC-MS) was performed on a Fisher Orbi-Trap Elite instrument (Thermo, Waltham, MA, United States) to detect and analyze the main constituents in DCHT.

### *In Vivo* Animal Study

**FIGURE 1:**
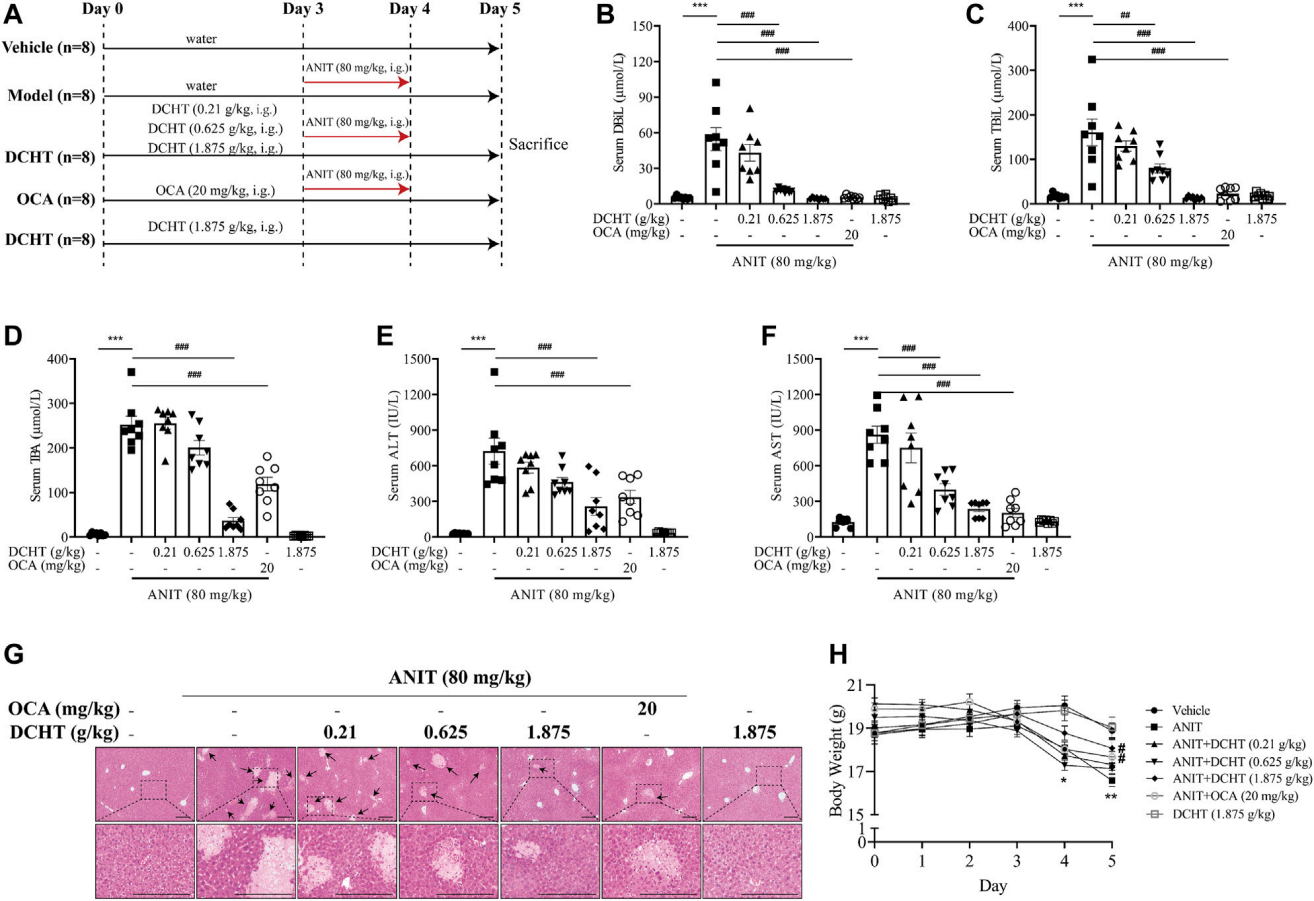
DCHT alleviates ANIT-induced cholestatic liver injury *in vivo*. **(A)** Scheme of the experimental design. **(B-F)** Serum levels of DBiL **(B)**, TBiL **(C)** and TBA **(D)**, ALT **(E)** and AST **(F)**. **(G)** H&E-stained liver sections. Scale bar = 200 μm. Arrows indicate the area of severe liver necrosis and hyperplastic bile cytoderm. **(H)** Body weights of all animals were recorded daily. Data are shown as means ± SEM; ^*^ *p* < 0.05, ^**^ *p* < 0.01, ^***^ *p* < 0.001 as compared with the vehicle group. ^#^ *p* < 0.05, ^##^ *p* < 0.01, ^###^ *p* < 0.001 as compared with the ANIT-treated group, *n* = 8.

Pathogen-free C57BL/6 male mice (8 weeks old, 18–20 g) were purchased from the Experimental Animal Center of Chinese Academy of Science (Shanghai, China). Experimental procedures were approved by the Shanghai University of Traditional Chinese Medicine Committee on the Use of Live Animals for Teaching and Research (Animal License: No. SYXK(HU)2014-0008; Registration number: PZSHUTCM190531014). To study intrahepatic cholestasis in response to α-naphthylisothiocyanate (ANIT), mice from administration groups were treated with DCHT [0.21, 0.625, and 1.875 g/kg dissolved in water, intragastric administration (i.g.)], or OCA (20 mg/kg dissolved in 0.5% CMC-Na, i. g., a mainstay treatment for cholestasis, as a positive control (Ding et al., 2018)), or PPARα antagonist GW6471 [5 mg/kg dissolved in 4% Tween 80 in saline (v/v), intraperitoneal administration (i.p.)] (Hu et al., 2019) or PPARα agonist fenofibrate (25 mg/kg dissolved in 0.5% CMC-Na, i. g. bid) (Dai et al., 2017) for 3 consecutive days. After treatment for 3 days, mice from each of the treatment groups ANIT, ANIT + DCHT, ANIT + OCA, ANIT + DCHT + GW6471, ANIT + GW6471, and ANIT + fenofibrate were given a single dose of ANIT (80 mg/kg dissolved in olive oil, i. g.), and mice from the control group were given the same volume of olive oil (i.g.) for two consecutive days (Figure 1A and Figure 6A). For extrahepatic cholestasis induced by bile duct ligation (BDL) as previous described (Tag et al., 2015), ligated mice were divided randomly into four groups (n = 6). The sham-operated control group (water, i. g.), BDL group (water, i. g.), and DCHT groups (0.21, 0.625, and 1.875 g/kg dissolved in water, i. g.) were given for 14 consecutive days (Supplementary Figure S2A). Animal body weight was recorded every day. After the treatment, blood samples were obtained by cardiac puncture, coagulated for 2 h at 4°C, and centrifuged at 3,000 × *g*, 4°C for 20 min. The activities of alanine aminotransferase (ALT), aspartate aminotransferase (AST), direct bilirubin (DBiL), total bilirubin (TBiL) and total bile acid (TBA) in the serum (supernatant) were measured using an automatic biochemistry analyzer Accute (TBA-40FR, TOSHIBA, Japan). Livers with gallbladders were excised from every mouse, and then either fixed in 4% paraformaldehyde (PFA) for analysis by Hematoxylin-Eosin (HE) staining or immediately frozen in liquid nitrogen and kept at −80°C until use for further analysis by Realtime PCR or SDS-PAGE/Western blotting.

### Measurement of Glutathione Content

Hepatic reduced glutathione (GSH) and oxidized glutathione (GSSG) were measured using the GSH and GSSG assay kit (Cat. #: S0053, Beyotime Inst. Biotech, Shanghai, China). The levels of GSH were calculated according to the difference between concentrations of total glutathione (GSH + GSSG) and GSSG. Hepatic levels of GSH were normalized to protein concentrations in the corresponding samples.

### Measurement of Enzymatic Activities

Hepatic activities of glutathione reductase (GR), glutamate-cysteine ligase (GCL), glutathione peroxidase (GSH-Px, GPX) and glutathione S-transferase (GST) were determined using the GR assay kit (Cat. #: A062), the GCL assay kit (Cat. #: A120-1-1), the GPX assay kit (Cat. #: A005), and the GST assay kit (Cat. #: A004), respectively. These assay kits were purchased from Nanjing Jiancheng Bioengineering Institute (Nanjing, China).

### Total RNA Extraction and Real-Time qPCR

Total RNA from the murine liver was extracted using the Trizol reagent (TaKaRa, Dalian, China). RNA concentrations were determined by nanodrop and then normalized. cDNA was synthesized from 1 µg of total RNA using the PrimeScript RT reagent Kit with gDNA Eraser (TaKaRa). qPCR reactions were performed using TB Green kit (TaKaRa) on an ABI 7500 real-time PCR system (Thermo Fisher, Quantstudio 3). Please see Supplementary Table S2 for a complete list of primers used in this study.

### SDS-PAGE/Western Blotting

Liver tissues were lysed in RIPA buffer containing 1 mM phenylmethylsulfonyl fluoride and protease inhibitor cocktail. Lysates were spun at 10,000 × *g* for 10 min and the supernatants kept at −20°C until use. Proteins were separated by SDS-PAGE and transferred to nitrocellulose filter membranes; blots were probed with appropriate combinations of primary and HRP-conjugated secondary antibodies. For repeated immunoblotting, membranes were stripped in 62.5 mM Tris (pH 6.7), 20% SDS and 0.1 M 2-mercaptoethanol for 30 min at 50°C prior to reprobing.

### Data Preparation and Network Construction

Putative targets of main constituents from DCHT based on the LC-MS based chemoprofile were obtained from Traditional Chinese Medicine Systems Pharmacology database (TCMSP) (http://tcmspw.com/tcmsp.php) (Ru et al., 2014) and SwissTargetPrediction (http://www.swisstargetprediction.ch/) (Gfeller et al., 2014). Known differentially expressed targets related to intrahepatic cholestasis and extrahepatic cholestasis were acquired from the GeneCards database (https://www.genecards.org/) (Safran et al., 2010) and DisGeNET database (https://www.disgenet.org/search) (Piñero et al., 2017). The visual network of the “Herb-Compound-Target-Disease” was established using Cytoscape software (version 3.7.2, Boston, MA, United States). The signaling pathways with enriched genes (FDR<0.05) were identified using database for Annotation, Visualization and Integrated Discovery (DAVID) assigning the Kyoto Encyclopedia of Genes and Genomes (KEGG) database.

### Dual Luciferase Reporter Gene Assay

HEK293T cells were cultured in Dulbecco’s Modified Eagle Medium (DMEM, Invitrogen) with 10% (v/v) fetal bovine serum (FBS, Invitrogen) without antibiotics in 96-wells plates (2 × 10^4^ cells/well) overnight, before co-transfection with a plasmid encoding PPARα (pCMX-Gal-mPPARα, 0.1 μg), report plasmid (pGL4-MH100 × 4-TK-Luc, 0.1 μg), and Renilla luciferase plasmid (pREP7, 0.01 μg) using Lipofectamine 3,000 (Invitrogen, Carlsbad, CA) as previous described (Huang et al., 2006). After treatment with DCHT or compounds, cells were collected and lysed. Firefly luciferase activity or Renilla luciferase activity (as normalization control) were determined using the Firefly & Renilla luciferase Reporter Assay Kit (Dalian Meilun Co., Ltd., China) and the BioTek Synergy™ 4 microplate reader (BioTek). Data were presented as firefly/renilla luciferase ratios. This allows the data to be normalized to control for factors such as transfection efficiency or overall translational activity.

### Statistical Analysis

Data are presented as means ± SEM. Statistical analysis was performed by LSD-test following one- or two-way ANOVA for multiple comparisons. *p* values less than 0.05 were considered as statistically significant differences.

## Results

### LC-MS Profile of DCHT

LC-MS based chemoprofile of the methanol solution of DCHT, including positive ion mode and negative ion mode were shown in Supplementary Figure S1. The presence of the following constituents in DCHT of detection mode, formula, molecular weight and Area (Max.) (Supplementary Table S3): (+)-Catechin, Lactiflorin, Albiflorin, Paeoniflorin, Oxypaeoniflorin and Benzoylpaeoniflorin (from Bai Shao); Succinic acid (from Ban Xia); Kaempferol, Sainfuran, Quercetin, Isorhamnetin, Saikosaponin A and Saikosaponin B1 (from Chai Hu); Chrysophanol, Aloe-emodin, Emodin, Rhein, Emodin-3-methyl ether, Chrysophanols 1-O-glucoside, Chrysophanols 8-O-glucoside, Emodin 8-O-glucoside, Emodin-1-O-beta-d-glucopyranoside and Rhein 8-O-glucoside (from Da Huang); (S)-Coclaurine (from Da Zao); Baicalein, Wogonin, Oroxylin A, Dihydrooroxylin A, Panicolin, Rivularin, Skullcapflavone II, Wogonin-7-O-glucuronoside and Oroxylin A-7-O-glucuronoside (from Huang Qin); 6-Gingerol (from Sheng Jiang); Synephrine, Naringenin, Luteolin, Hesperetin, Tetramethoxyluteolin, Sinensetin, Isosinensetin, Nobiletin, Naringin, Narirutin, Hesperidin and Neohesperidin (from Zhi Shi), and Baicalin is shared by Chai Hu, Huang Qin and Ban Xia, and some major constituents from DCHT were the same as previously described (Yoshie et al., 2004; He et al., 2014).

### DCHT Treatment Alleviated Cholestatic Liver Injury in Response to ANIT

ANIT is metabolized by cytochrome P450 and is subjected to GSH conjugation, and thus induces intrahepatic cholestasis, biliary epithelial cell necrosis, bile duct obstruction and hepatocellular injury, such characteristics mimic the drug-induced cholestasis and hepatic injury in humans (Rodríguez-Garay, 2003). We first established an acute cholestatic murine model by treating mice with ANIT for 48 h, and used OCA (20 mg/kg, a mainstay treatment for cholestasis) as a positive control (Figure 1A) (Ding et al., 2018). At the end of this treatment period, we observed an increase in the levels of indicators of cholestasis, namely DBiL and TBiL, in response to ANIT (Figures 1B,C). In addition, TBA, a typical indicator of intrahepatic cholestasis, was elevated after ANIT treatment (Figure 1D). The induction of serum DBiL, TBiL and TBA by ANIT was effectively reversed by DCHT treatment in a dose-dependent manner (Figures 1B–D). After the treatment of ANIT, serum levels of ALT and AST, which serve as direct indicators of liver injury, were elevated (Figures 1E,F), accompanied by large periportal hemorrhage, diffuse vacuolization, inflammatory infiltration, and parenchymal necrosis in the liver (Figure 1G). DCHT treatment was able to alleviate ANIT-induced liver injury in a dose-dependent manner, which is similar to the effect of OCA (20 mg/kg) (Figures 1E–G). ANIT also caused a dramatic loss of body weight, which was attenuated upon treatment with DCHT (1.875 g/kg) or OCA (Figure 1H). However, DCHT treatment never alleviated BDL-induced extrahepatic cholestasis with liver injury (Supplementary Figure S2). These data indicate that DCHT can protect mice from ANIT-induced acute intrahepatic cholestasis with liver injury.

### DCHT Treatment Inhibited Bile Accumulation by Restoring the Gene Expression Profile Implicated in Bile Acid Homeostasis

**FIGURE 2:**
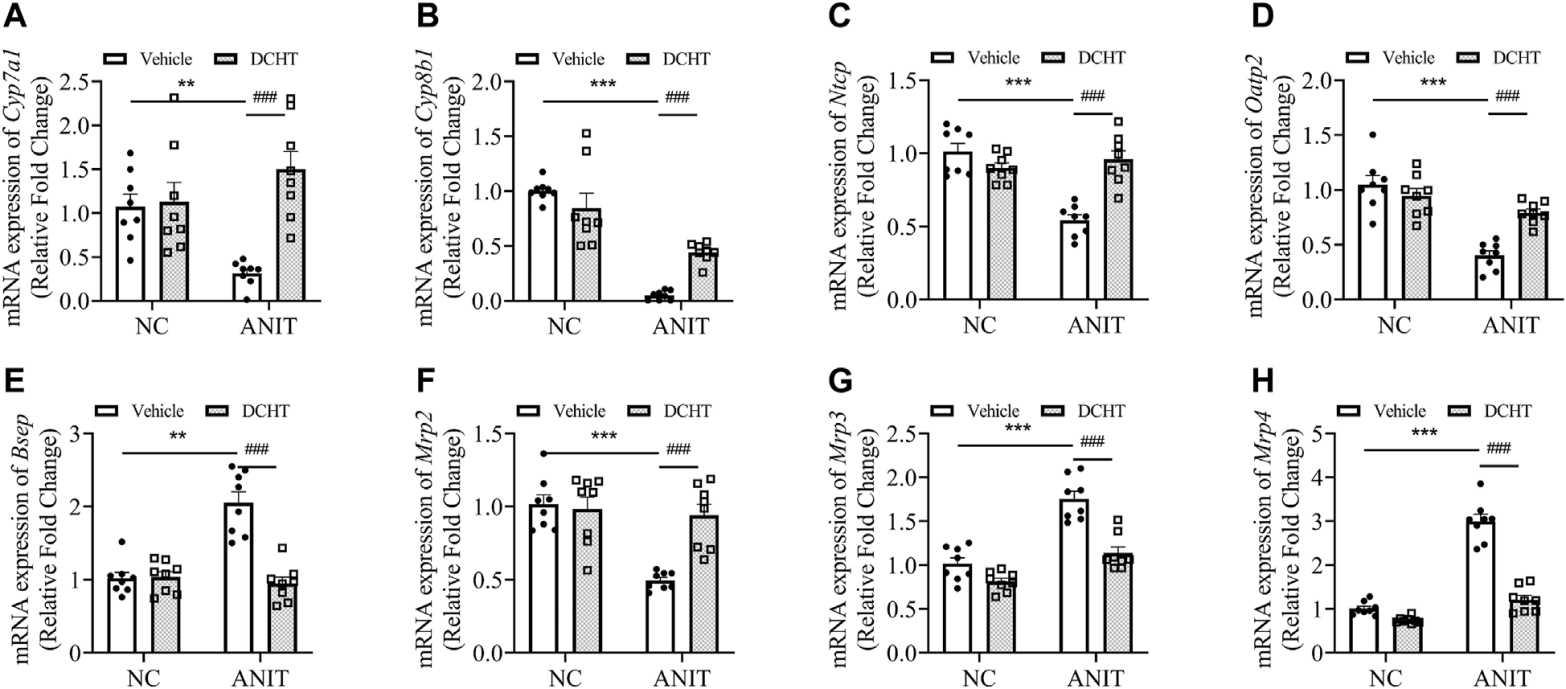
DCHT alters hepatic mRNA expression of genes related to bile acid homeostasis in ANIT-induced intrahepatic cholestasis *in vivo*. **(A-H)** Hepatic mRNA expression of *Cyp7a1* **(A)**, *Cyp8b1* **(B)**, *Ntcp* **(C)**, *Oatp2* **(D)**, *Bsep* **(E)**, *Mrp2* **(F)**, *Mrp3* **(G)** and *Mrp4* **(H)** was measured by qPCR after treatment of mice with ANIT (2 days) and/or DCHT (5 days, 1.875 g/kg), as indicated. Data are shown as means ± SEM.; ^**^ *p* < 0.01, ^***^ *p* < 0.001 compared with the vehicle group; ^###^ *p* < 0.001 compared with the ANIT treatment group, *n* = 8.

To investigate whether DCHT modulates bile acid homeostasis during acute intrahepatic cholestasis, we treated the mice with 1.875 g/kg DHCT and then examined the expression of genes implicated in hepatic bile acid biosynthesis, uptake and efflux. As shown in Figures 2A,B, the expression of the mRNA encoding the cytochromes cholesterol 7α-hydroxylase (*Cyp7a1*) and sterol 12α-hydroxylase (*Cyp8b1*) was down-regulated in response to ANIT treatment and restored by DCHT. Bile acids are first taken up by the hepatobiliary transporters NTCP and OATP2 from the plasma and are then subsequently exported into the bile by the canalicular transporters BSEP and MRP2. ANIT treatment led to a decrease in the mRNA levels for *Ntcp* and *Oatp2*, and this reduction was prevented by DCHT (Figures 2C,D). ANIT treatment also increased the mRNA levels for *Bsep* and decreased the level of *Mrp2*, which encode for bile acid efflux transporters BSEP and MRP2, respectively; both of these effects were reversed by DCHT (Figures 2E,F). Multidrug resistance-associated protein 3 (MRP3) and multidrug resistance-associated protein 4 (MRP4) are transporters of the hepatocyte’s basolateral membrane with a compensatory role. Both MRP3 and MRP4 transporters’ increased mRNA expression plays an essential role in the protective and adaptive responses of bile acid overload, and the induction of mRNA level for *Mrp3* and *Mrp4* was effectively reversed by DCHT treatment (Figures 2G,H). These results suggest that DCHT protects against acute intrahepatic cholestasis by restoring bile acid homeostasis.

### DCHT Treatment Alleviated Liver Injury by Restoring Glutathione Homeostasis and Inhibiting Hepatic Inflammation in ANIT-Induced Acute Cholestatic Mice

**FIGURE 3:**
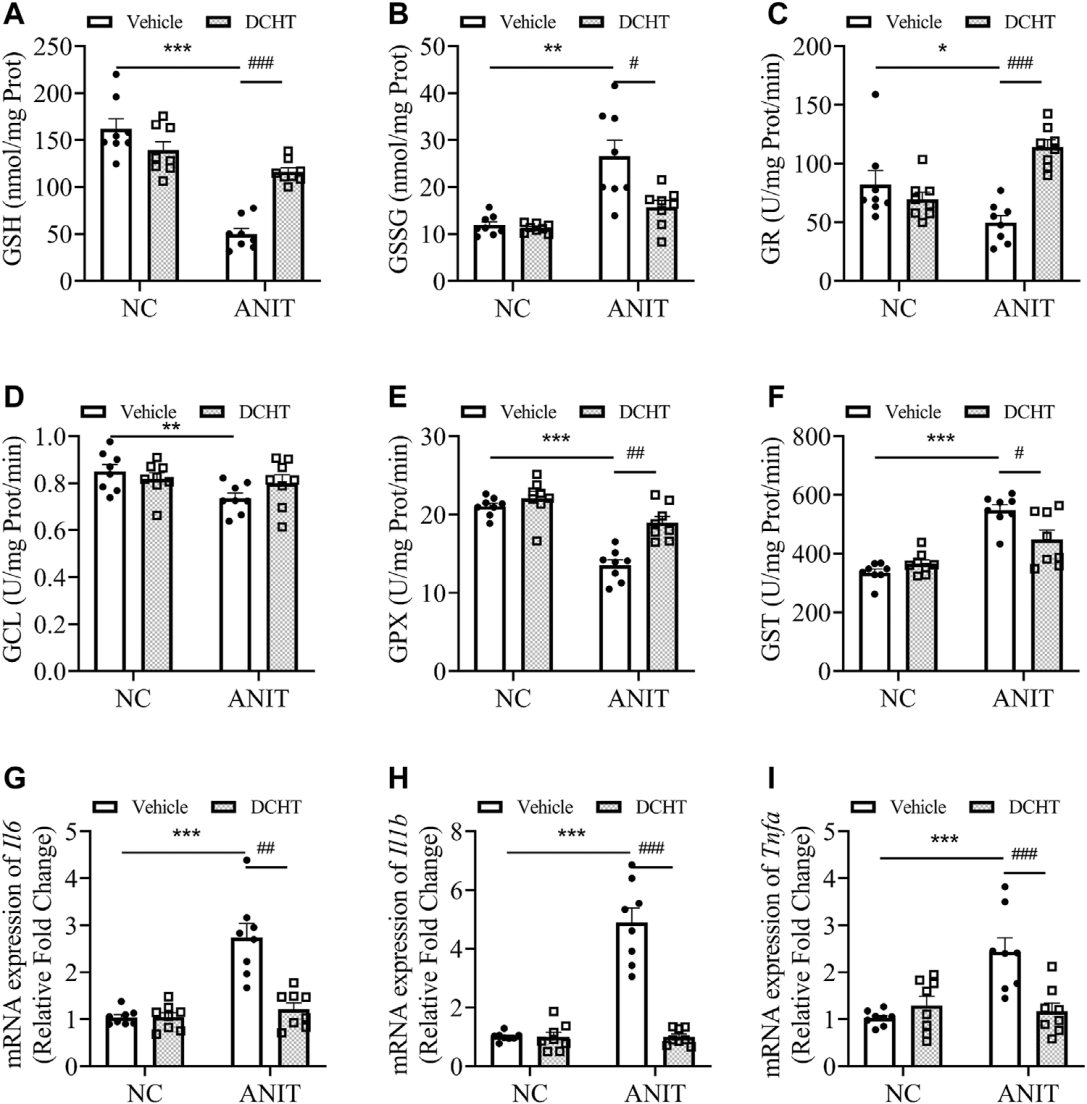
DCHT regulates hepatic glutathione homeostasis and inflammation in ANIT-induced intrahepatic cholestasis mice. **(A-F)** Hepatic activities of GSH **(A)**, GSSG **(B)**, GR **(C)**, GCL **(D)**, GPX **(E)** and GST **(F)** in the liver were measured after treatment of mice with ANIT (2 days) and/or DCHT (5 days, 1.875 g/kg), as indicated **(G-I)** mRNA expression of *Il6* **(G)**, *Il1b* **(H)** and *Tnfa* **(I)** in the liver was measured by RT-qPCR after treatment of mice with ANIT (2 days) and/or DCHT (5 days, 1.875 g/kg), as indicated. Data are shown as means ± SEM.; ^*^ *p* < 0.05, ^**^ *p* < 0.01, ^***^ *p* < 0.001 compared with the vehicle group. ^#^ *p* < 0.05, ^##^ *p* < 0.01, ^###^ *p* < 0.001 compared with the ANIT treatment group, *n* = 8.

Accumulated toxic bile acids led to oxidative stress and then caused liver injury, which can be alleviated by glutathione *via* eliminating reactive oxygen species (ROS) (Orozco-Aguilar et al., 2021). Indeed, GSH (reduced glutathione) levels were decreased upon ANIT treatment whereas GSSG (oxidized glutathione) was elevated, leading to a marked decrease in the ratio of GSH/GSSG, an effect which was also prevented by DCHT (Figures 3A,B). DCHT treatment led to a remarkable restoration of GR activity, whereas had little effect on GCL activity in the presence of ANIT (Figures 3C,D). The inhibition of GPX activity by ANIT was abolished by DCHT *in vivo* (Figure 3E). Moreover, while the activity of the detoxifying enzyme GST was increased in response to ANIT, it was reduced by DCHT in combination with ANIT treatment (Figure 3F). Interleukin-6 (IL-6), Interleukin-1ß (IL-1ß) and tumor necrosis factor alpha (TNF-α) participate in the progression in cholestatic liver injury (Bode et al., 2012). We detected an increase in *Il6*, *Il1b* and *Tnfa* mRNA expression, in liver tissue derived from the ANIT-treated mice compared to the control ones, and this effect was reversed in the ANIT + DCHT group of mice (Figures 3G–I), thus indicating that DCHT treatment was able to suppress the mRNA expression of hepatic inflammatory markers induced by ANIT. Taken together, these results indicate that DCHT is capable of alleviating acute cholestasis-induced liver damage by inhibiting hepatic inflammation and restoring glutathione homeostasis.

### MAPK/NF-ĸB/STAT3 Signaling Axes Were Inactivated Upon DCHT Treatment in the Setting of Intrahepatic Cholestasis With Liver Injury in Response to ANIT

**FIGURE 4:**
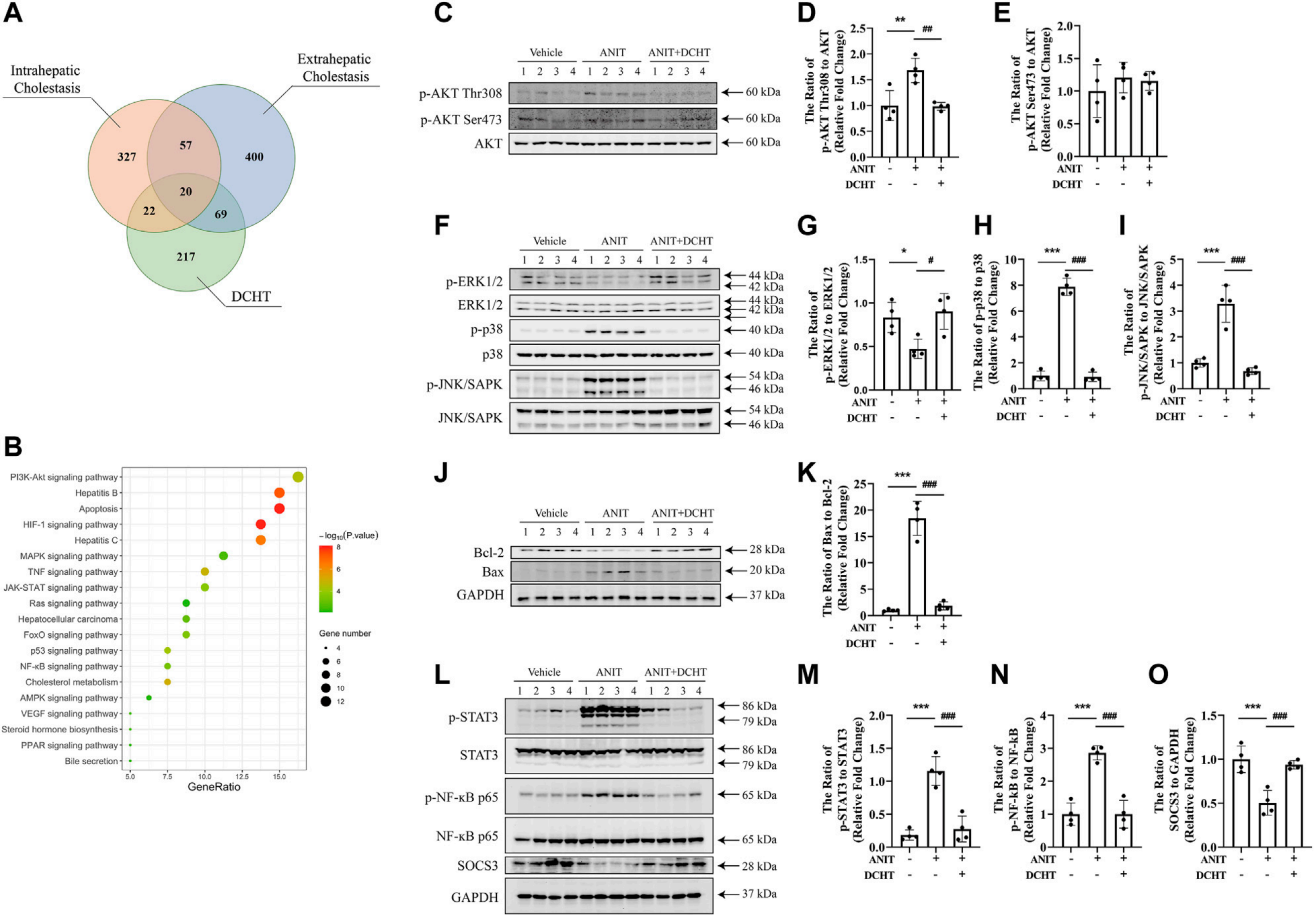
DCHT regulates the signaling axes of MAPK/NF-ĸB/STAT3 in ANIT-induced cholestatic liver injury *in vivo*. **(A)** Venn diagram of putative targets in DCHT and cholestasis. The orange circle, blue circle and green circle indicate 426 putative targets involved in intrahepatic cholestasis, 546 putative targets involved in extrahepatic cholestasis and 328 putative targets involved in DCHT treatment, respectively. **(B)** KEGG pathway enrichment analysis of the DCHT targets from Figure 5A. **(C–O)** Protein levels of p-AKT Thr308, p-AKT Ser473, AKT, p-ERK1/2, ERK, p-p38, p38, p-JNK/SAPK, JNK/SAPK, Bcl-2, Bax, p-STAT3, STAT3, p-NF-ĸB p65, NF-ĸB p65, SOCS3 and GAPDH were measured by immunoblot after DCHT treatment (1.875 g/kg). The data are expressed as the ratios of p-AKT Thr308 to AKT **(D)**, p-AKT Ser473 to AKT **(E),** p-ERK1/2 to ERK **(G)**, p-p38 to p38 **(H)**, p-JNK/SAPK to JNK/SAPK **(I)**, Bax to Bcl-2 **(K)**, p-STAT3 to STAT3 **(M)**, p-NF-ĸB p65 to NF-ĸB p65 **(N)**, SOCS3 to GAPDH **(O)**. Data are shown as means ± S.E.M.; ^*^ *p* < 0.05, ^**^ *p* < 0.01, ^***^ *p* < 0.001 compared with control group; ^#^ *p* < 0.05, ^##^ *p* < 0.01, ^###^ *p* < 0.001 compared with ANIT treatment group, *n* = 4.

To determine the molecular mechanisms by which DCHT exerts its protective effect on acute intrahepatic cholestasis induced-liver injury, we exploited the GeneCards and DisGeNET databases which identified 42 putative targets involved in DCHT treatment for intrahepatic cholestasis (Figure 4A and Supplementary Table S4) and applied KEGG pathway enrichment analysis to the DCHT target data (Figure 4B). Notably, the expression of genes implicated in the PI3K-Akt signaling pathway was the most affected by ANIT treatment (Figure 4B). As a validation for our pathway enrichment analysis, we did indeed observe a slight increase in the level of AKT phosphorylation at Thr308 but with no change at Ser473 (Figures 4C–E); a modest decrease in the level of ERK1/2 phosphorylation (Figures 4F,G); an increase in the phosphorylation of p38 MAP kinase (Figures 4F,H) and JNK/SAPK (Figures 4F,I) in the livers of mice treated with ANIT. These effects were reversed by DCHT co-treatment. We also observed a decrease in Bcl-2 expression and an increase in the expression of Bax in ANIT-treated mice, leading to an increase in the proapoptotic/antiapoptotic Bax/Bcl-2 ratio. The proapoptotic effect of ANIT in the liver tissues was attenuated upon DCHT treatment (Figures 4J,K), consistent with the protective effect of DCHT on liver injury (Figure 1G). Western blot analysis demonstrated that STAT3 was strongly phosphorylated in the livers of mice treated with ANIT, and this was prevented by DCHT co-treatment (Figures 4L,M). Similarly, DCHT treatment effectively antagonized the phosphorylation of NF-ĸB in response to ANIT (Figures 4L–N). Additionally, DCHT treatment prevented the ANIT-induced decrease in expression of the SOCS3 protein, a feedback inhibitor of the STAT3 signaling pathway which also reduces NF-ĸB activity (Gu et al., 2011) (Figures 4L–O). This implies that the suppression of MAPK/NF-ĸB/STAT3 signaling axes may contribute to the preventative effect of DCHT on ANIT-induced liver injury.

### PPARα Activation Plays a Key Role in the DCHT Treatment for Intrahepatic Cholestasis

**FIGURE 5:**
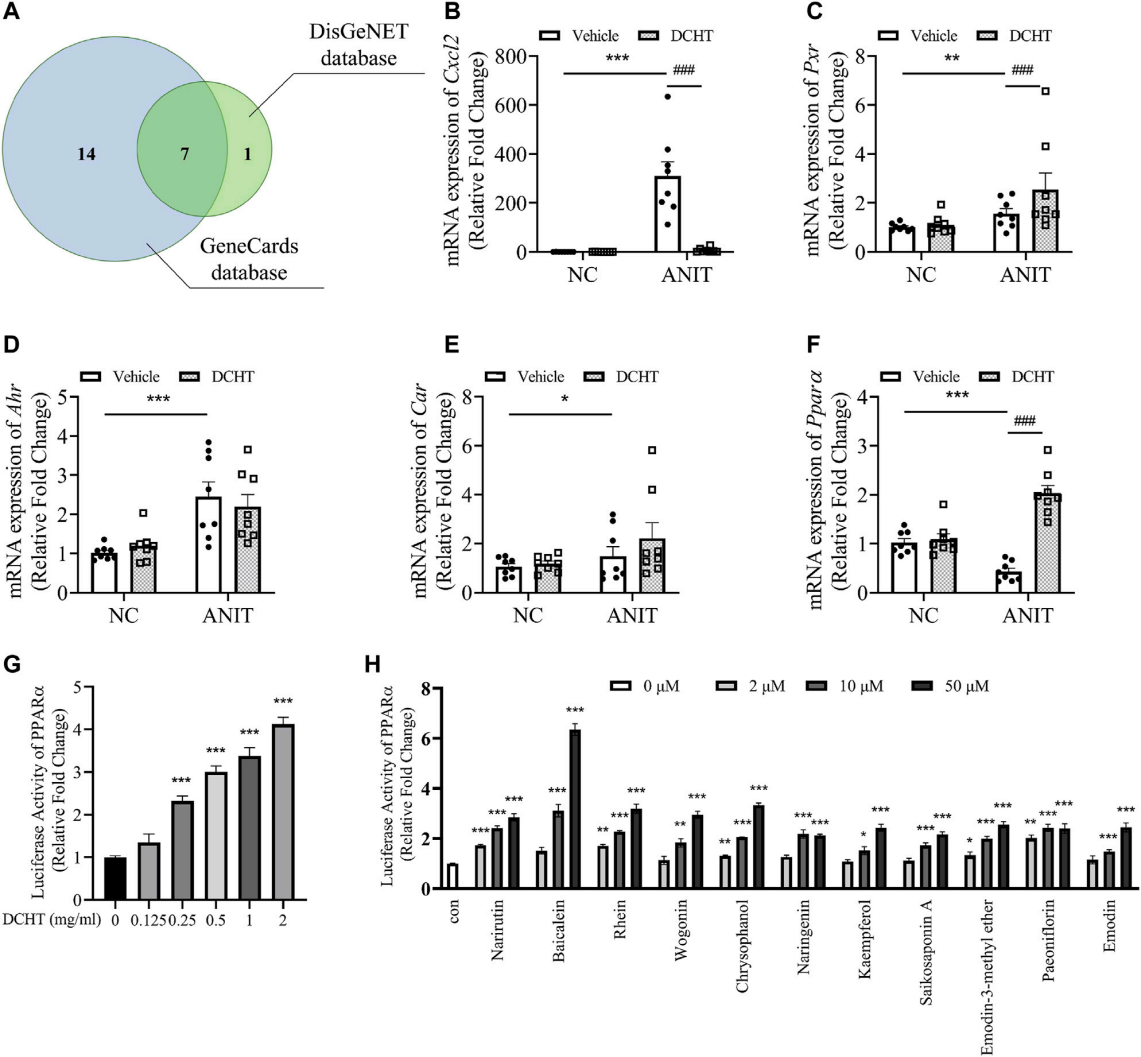
PPARα is a potential target involved in the DCHT treatment for intrahepatic cholestasis. **(A)** Venn diagram of 22 putative targets of DCHT acting on intrahepatic cholestasis not extrahepatic cholestasis from Figure 5A. Putative targets in the blue and green circles are from GeneCards database and DisGeNET database, respectively. **(B-F)** Hepatic mRNA expression of *Cxcl2* **(B)**, *Pxr* **(C)**, *Ahr* **(D)**, *Car* **(E)** and *PPARa* **(F)** was measured by RT-qPCR after treatment of mice with ANIT (2 days) and/or DCHT (5 days, 1.875 g/kg), as indicated. Data are shown as means ± SEM.; ^*^ *p* < 0.05, ^**^ *p* < 0.01, ^***^ *p* < 0.001 compared with the vehicle group; ^###^ *p* < 0.001 compared with the ANIT treatment group, *n* = 8. **(G,H)** luciferase activity from a PPARα-driven luciferase reporter was determined after treatment of HEK293T cells with DCHT (0–2 mg/ml) **(G)** or indicated compounds (2–50 μM) **(H)** for 24 h using dual-luciferase reporter approach. Data are shown as means ± SEM; ^*^ *p* < 0.05, ^**^ *p* < 0.01, ^***^ *p* < 0.001 compared with control, *n* = 3.

Among these genes affected by DCHT as discovered by network pharmacology, 22 were predicted to be associated with intrahepatic but not extrahepatic cholestasis (Figure 5A and Supplementary Table S4). Among those 22 genes, 7 genes (*PPARα*, *CXCL2*, *PXR*, *CAR*, *AHR*, *ESR1* and *ESR2*) were identified from both the GeneCards and the DisGeNET databases (Figure 5A and Supplementary Table S5). We validated the changes in 5 out of 7 core putative targets in the treatment of DCHT for ANIT-induced intrahepatic cholestasis, with the exception of estrogen receptor (ESR) 1 and 2 (*ESR1* and *ESR2*) because they are more associated with intrahepatic cholestasis during pregnancy (Song et al., 2014). As shown in Figure 5B, the expression of the mRNA encoding the cytochromes C-X-C motif chemokine ligand 2 (*Cxcl2*, encoded by CXCL2) was upregulated in response to ANIT treatment and suppressed by DCHT. The expression of the mRNA for Pregnane X receptor (*Pxr*, encoded by NR1I2) was upregulated in response to ANIT treatment and further enhanced by DCHT (Figure 5C). DCHT treatment had no effect on the large increases in the expression of the mRNAs for both the aryl hydrocarbon (*Ahr*, encoded by AHR) and constitutive androstane receptors (*Car*, encoded by NR1I3) in response to ANIT (Figures 5D,E). Of note, ANIT treatment led to a decrease in the mRNA expression of *Ppara* (encoded by NR1C1) compared to the vehicle-treated group; and this effect was ameliorated in response to DCHT (Figure 5F). We also noted that there was a dose-dependent increase in the activity of PPARα after DCHT treatment for 24 h as determined by a dual-luciferase reporter gene assay (Figure 5G). In addition, as evidenced by the dual-luciferase reporter gene assay, any of these 11 compounds was able to increase PPARα activity individually in a dose-dependent manner (Figure 5H), which is similar to the effect of fenofibrate, a classic agonist of PPARα (Ghonem et al., 2015) (Supplementary Figure S3).

**FIGURE 6:**
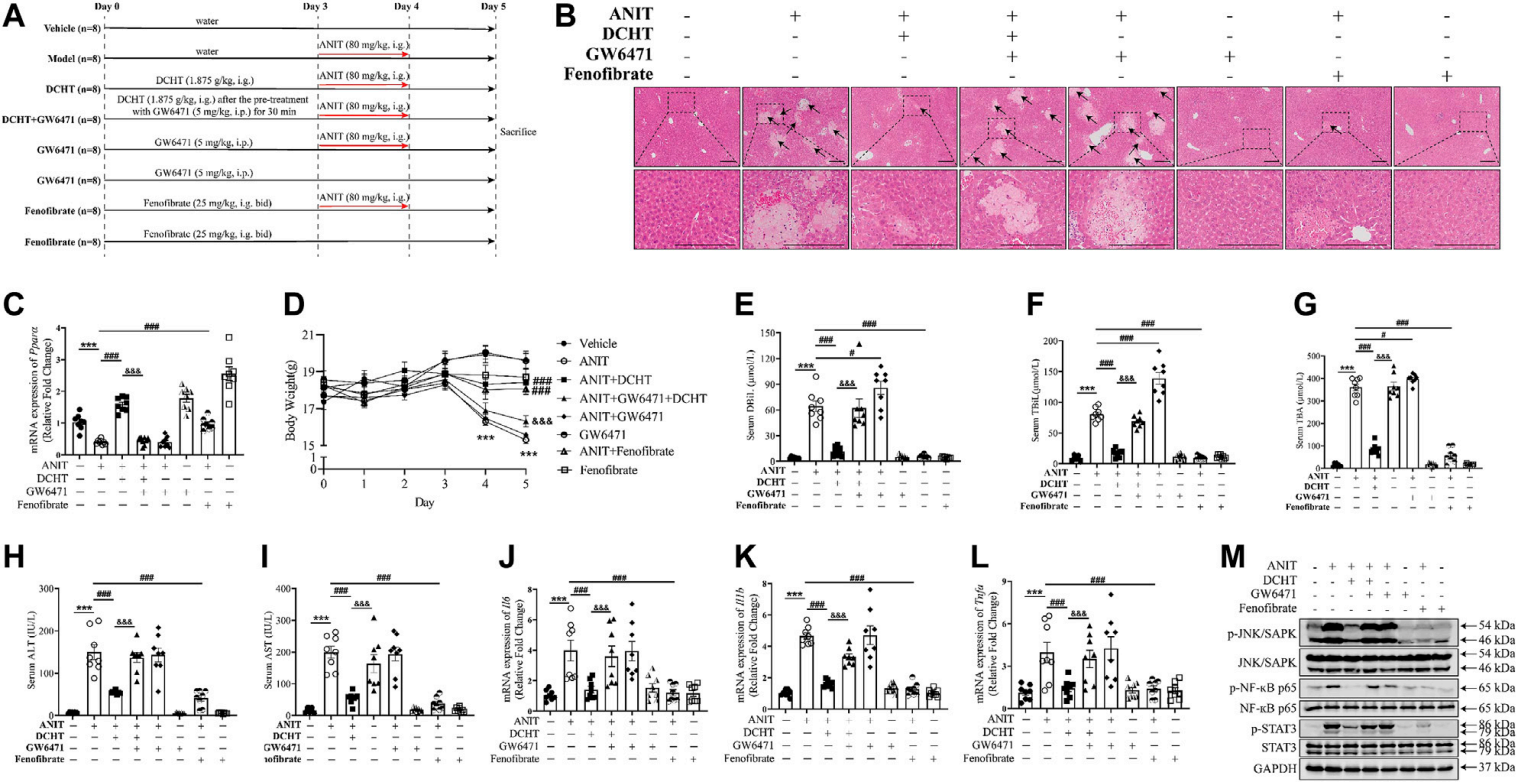
DCHT protects against intrahepatic cholestasis *via* activation of PPARα. **(A)** Scheme of the experimental design to study the effect of DCHT in combination with PPARα antagonist GW6471, or the PPARα antagonist GW6471, or the PPARα agonist fenofibrate in response to ANIT. Mice were treated with ANIT (2 days) and/or DCHT (5 days, 1.875 g/kg), GW6471 (5 days, 5 mg/kg) and fenofibrate (5 days, 25 mg/kg, bid) as indicated. **(B)** H&E-stained liver sections. Scale bar = 200 μm. Arrows indicate the area of severe liver necrosis and hyperplastic bile cytoderm. **(C)** Hepatic expression of *Ppara* mRNA was measured by RT-qPCR. **(D)** Body weights of all animals were recorded daily. **(E-I)** Serum levels of DBiL **(E)**, TBiL **(F)** and TBA **(G)**, ALT **(H)** and AST **(I)**. **(J-L)** expression of the *Il6* **(J)**, *Il1b* **(K)** and *Tnfa* **(L)** mRNAs in the liver was measured by RT-qPCR **(M)** Protein levels of p-JNK/SAPK, JNK/SAPK, p-STAT3, STAT3, p-NF-ĸB p65, NF-ĸB p65 and GAPDH were measured by immunoblot. Data are shown as means ± SEM.; ^***^ *p* < 0.001 ANIT treatment group compared with the vehicle group; ^#^ *p* < 0.05, ^###^ *p* < 0.001 compared with the ANIT treatment group; ^&&&^ *p* < 0.001 ANIT + GW6471 + DCHT group compared with ANIT + DCHT group, *n* = 8.

To gain insight into the role of PPARα in DCHT treatment for cholestatic liver injury, we assessed the effect of DCHT, in combination with PPARα antagonist GW6471 or PPARα agonist fenofibrate, in response to ANIT. Fenofibrate protected against ANIT-induced cholestasis with liver injury, as demonstrated by the disappearance of tissue necrosis. Serum levels of DBiL, TBiL, TBA, ALT and AST were reduced in fenofibrate-treated mice (Dai et al., 2017), a similar effect was also observed in the DCHT treatment group (Figures 6A–I). In contrast, GW6471 treatment accelerated cholestasis and had no protective effect on liver injury in response to ANIT (Figures 6A–I). Of note, pre-treatment with GW6471 attenuated the protective effect of DCHT on cholestatic liver injury evoked by ANIT (Figures 6A–I). The protective effects of fenofibrate and DCHT on ANIT-induced cholestasis and liver injury coincident with the inhibition of mRNA expression of inflammation-related genes (*Il6*, *Il1b*, and *Tnfa*), as well as the suppression of JNK/SAPK, NF-ĸB and STAT3 pathways. Such effects could be reversed upon GW6471 treatment (Figures 6J–M). Taken together, these data provide compelling evidence that PPARα is a potential target of DCHT in the treatment of intrahepatic cholestasis with liver injury.

## Discussion

In the present study, we found that DCHT was able to greatly alleviate acute intrahepatic cholestasis and protect against cholestasis-induced liver injury without any apparent toxicity *in vivo via* PPARα activation, thus providing strong evidence that DCHT is a potentially useful therapeutic formula for the prevention and treatment of intrahepatic cholestatic hepatoxicity.

Failure of biliary bile acid excretion during both intrahepatic cholestasis and extrahepatic cholestasis results in the retention and accumulation of hydrophobic bile acids in the liver. The accumulation of toxic bile acids inside hepatocytes is one of the main causes of cholestasis-induced liver damage, a process which is characterized by ALT and AST leaking out from the cytosol into the blood stream, plus structural and functional injuries to hepatocyte membranes, and ultimately, liver cell death (Li and Apte, 2015). The present findings provide compelling evidence to support a protective role of DCHT in ANIT-induced intrahepatic cholestasis with liver injury, as demonstrated by the changes of gallbladder and liver histopathological morphology, aforementioned enzymatic indicators, the phosphorylation of p38 MAP kinase, the dephosphorylation of ERK1/2, as well as an increase in the proapoptotic/antiapoptotic (Bax/Bcl-2) ratio in the liver tissue. However, DCHT treatment has no effect on extrahepatic cholestasis in response to BDL.

To our knowledge, this is the first report to identify PPARα as a crucial factor implicated in DCHT treatment for intrahepatic cholestasis. PPARα, as a ligand-activated transcription factor that is abundantly expressed in liver, has a complicated role in biliary phospholipid secretion, bile acid metabolism and bile acid synthesis (Ghonem et al., 2015). A combination of PPARα agonist fenofibrate and UDCA could decrease serum AST, ALT, ALP, γ-glutamyl transpeptidase, and TG in PBC (classic intrahepatic cholestasis) patients, who are not responsive to UDCA alone (Ghonem and Boyer, 2013). ANIT can be metabolized by CYP450 enzymes (Gallenkamp and Richter, 1974), among which *Cyp7a1* controls the rate of hepatic bile acid synthesis (Chiang, 2009) and hepatic *Cyp8b1* regulates the ratio of cholic acid (CA) to chenodeoxycholic acid (CDCA) in the bile acid pool (Li and Apte, 2015). The activity of PPARα is inhibited in response to ANIT treatment (Dai et al., 2018), whereas PPARα agonist Wy-14643 was also able to induce the transcription of *Cyp7a1* and *Cyp8b1*, as well as increased their enzymatic activities, resulting in altered bile acid homeostasis in the presence of ANIT (Hunt et al., 2000; Marrapodi and Chiang, 2000). In this study, DCHT treatment restored the reduced expression of the mRNAs *Cyp7a1* and *Cyp8b1*, members of the intrahepatic cholestatic group *via* activation of PPARα.

NTCP and OATP2, as basolateral domain hepatic bile acid transporters, are responsible for reabsorption of bile acid from the portal blood supply into the hepatocytes (Dawson et al., 2009). We found that ANIT downregulated *Ntcp* and *Oatp2* mRNA levels in murine liver tissues, and this was also restored by DCHT. BSEP, a bile flow pump situated on the cholesterol-rich canalicular membranes of hepatocytes, is mainly responsible for eliminating unconjugated and conjugated bile acids/salts from hepatocytes into the bile duct (Telbisz and Homolya, 2016). Surprisingly, ANIT evoked an increase in *Bsep* mRNA levels, suggesting the induction of an adaptive response that protects against the excessive hepatic accumulation of toxic bile acids. DCHT treatment caused a decrease in *Bsep* mRNA levels in response to ANIT, similar to the effect of PPARα agonist Wy-14643 (Xie et al., 2019). MRP2 is an important hepatic canalicular transporter that mediates the efflux of bile acids, conjugated bilirubin and GSH (Dietrich et al., 2001). Expression of MRP3 and MRP4 is induced under cholestatic conditions, and/or where MRP2 function is impaired, functioning as an alternative hepatocellular protection pathway when normal canalicular bile salt transport is compromised (Siewert et al., 2004; Geier et al., 2007). DCHT treatment reversed the reduction in *Mrp2* mRNA expression and inhibited the increase in mRNA expression of *Mrp3* and *Mrp4* that occurred in response to ANIT. Taken together, the observed modulation of bile acid transporter expression may contribute to the capacity of DCHT to “clean up” accumulated toxin bile acids.

Accumulated toxic bile acids led to oxidative stress, which can be alleviated by glutathione *via* elimination of reactive oxygen species (ROS) (Orozco-Aguilar et al., 2021). Glutathione homeostasis is modulated by adjusting the balance between the synthesis, utilization and recycling of glutathione (Lushchak, 2012). PPARα deficiency contributes to ROS production, and inhibition of PPARα may increase the susceptibility of the liver tissues to organ damage in the presence of another toxic agent (Abdelmegeed et al., 2009). The present study showed that ANIT treatment inhibited PPARα activity, and disturbed glutathione homeostasis by decreasing reduced glutathione content and increasing the level of oxidized glutathione, indicative of oxidative stress. PPARα, as an antioxidant, is known to protect the liver tissues against acetaminophen-mediated toxicity (Shankar et al., 2003). Here, we found DCHT treatment not only leads to an increase in the mRNA level of PPARα but also its activity. Therefore, activation of PPARα by DCHT may protect against intrahepatic cholestatic liver injury.

Consistent with an increase in the levels of hepatic GSH, DCHT treatment rescued the reduction in GR and GPX (an antioxidant) activities (Lu, 2013; Scirè et al., 2019) that were depleted in ANIT-induced intrahepatic cholestasis. GST is an important detoxifying enzyme which catalyzes the conjugation of reduced GSH with electrophilic endogenous and xenobiotic compounds, and subsequently converts them to less toxic water-soluble products which can then be eliminated from the cell (Lu, 2013). GST activity was elevated after the treatment with ANIT, and this was reversed by co-treatment with DCHT.

Hepatic JNK/SAPK deficiency suppressed the activation of the transcription factor PPARα to alter bile acid homeostasis (Manieri et al., 2020). The PPARα agonist Wy-14643 attenuated ANIT-induced cholestasis and liver injury, coincident with the inhibition of JNK signalling (Dai et al., 2017). The accumulation of NF-κB in response to the activation of JNK/SAPK is a pivotal factor that transmits inflammatory signals from the cytoplasm into the nucleus to promote the release of pro-inflammatory cytokines, such as IL-6, IL-1β and TNF-α, from hepatocytes (Karin, 2006). IL-6 induced by IL-1β *via* PI3K-AKT axis directly activates STAT3 and stimulates hepatocyte apoptosis, leading to aggravated liver damage (Cahill and Rogers, 2008; Bode et al., 2012). The expression of SOCS3 is controlled by both NF-κB and STAT3 and may act as a signaling link between these two pathways (Grivennikov and Karin, 2010). In particular, SOCS3 binds to the gp130 receptor subunit and prevents further cytokine-dependent activation of STAT3 (Babon et al., 2014). Likewise, SOCS3 can also attenuate the activation of NF-κB that is driven by several cytokines and Toll-like receptor (TLR) agonists (Grivennikov and Karin, 2010). Given that STAT3 prolongs nuclear retention of NF-κB, SOCS3-mediated inactivation of STAT3 may also be responsible for, or contribute to, the reduction in NF-κB activity in response to upstream signaling cues (Gu et al., 2011). Activation of PPARα can induce an increase in IκBα mRNA and protein levels to negatively regulate NF-κB, and consequently prevent the generation of inflammatory cytokines, such as IL-1ß, IL-6 and TNF-α, and thus inhibit the activation of STAT3 to protect the liver from cholestasis (Delerive et al., 1999; Delerive et al., 2000; Ghonem et al., 2015; Christofides et al., 2021), which agrees with our data showing that ANIT-induced cholestatic liver injury was associated with a deficiency in *PPARα* expression, the generation of *Il6* and the activation of NF-κB/STAT3 axis; whereas DCHT can reverse such effects. Indeed, a previous study has demonstrated that the activation of NF-κB/STAT3 signaling is responsible for inducing liver injury in *Pparα*-null mice (Dai et al., 2018). Therefore, it is plausible that PPARα acts as an upstream negative regulator of STAT3, and ANIT may induce the phosphorylation of STAT3 by reducing *Pparα* and subsequently increasing *Il6* levels. In addition, PPARα antagonist GW6471 attenuated the protective effect of DCHT on intrahepatic cholestasis consistent with the inhibition of the JNK/SAPK, NF-ĸB and STAT3 signaling pathways and the mRNA levels of IL-6, IL-1β and TNF-α, implying that PPARα is very likely to contribute to the anti-inflammation response and the cytoprotective effect of DCHT on acute intrahepatic cholestasis.

Besides cholestasis, PPARα is also involved in the treatment of obesity-related diseases, such as atherosclerosis and non-alcoholic fatty liver disease (Pawlak et al., 2015). Our network pharmacology-based approach established PPARα as the core element of DCHT’s visual Herb-Compound-Target-Disease network (Supplementary Figure S4). This may explain why DCHT is considered as a treatment of obesity and hyperlipidemia (Umeda et al., 1989; Han et al., 2020), since PPARα plays a central role in the onset and progression of these diseases (Xu et al., 2018). Whether DCHT is a direct or indirect PPARα agonist awaits further investigation. Besides, the protective effect of DCHT on intrahepatic cholestasis with hepatotoxicity should be further explored in PPARα-null mice; or in the clinic, i.e., to be tested on intrahepatic cholestatic patients, and ultimately leading to the development of a new therapeutic strategy to treat intrahepatic cholestasis not extrahepatic cholestasis.

In conclusion, our study has provided compelling evidence that DCHT, as a potential PPARα agonist, can alleviate acute intrahepatic cholestasis with liver injury by reversing disordered bile acid and glutathione homeostasis, and inhibiting inflammatory cytokines, with the JNK/NF-ĸB/IL-6/STAT3 signaling cascades concurrently participating in the process (Supplementary Figure S5). To our knowledge, this is the first study to “holistically” elucidate the pharmacological mechanisms of DCHT, a well-established TCM formula in the clinic, from molecular, cellular, organismal, and systematical perspectives, and thus pave the way for a better understanding of the pharmacology of traditional Chinese medical formulae.

## Data Availability

The original contributions presented in the study are included in the article/Supplementary Material, further inquiries can be directed to the corresponding authors.

## Glossary

ANIT: α-naphthylisothiocyanate
AHR: aryl hydrocarbon receptor
ALT: alanine aminotransferase
AST: aspartate aminotransferase
BDL: bile duct ligation
BSEP: bile acids export pump
CAR: Constitutive androstane receptor
CXCL2: C-X-C motif chemokine ligand 2
Cyp7a1: cholesterol 7α-hydroxylase
Cyp8b1: sterol 12α-hydroxylase
DAVID: database for annotation, visualization and integrated discovery
DBiL: direct bilirubin
DCHT: Da-Chai-Hu-Tang
DMSO: absolute dimethyl sulfoxide
ESR: estrogen receptor
FBS: fetal bovine serum
FDA: food and drug administration
FXR: farnesoid X nuclear receptor
GCL: glutamate-cysteine ligase
GR: glutathione reductase
GSH-Px, GPX: glutathione peroxidase
GSSG: oxidized glutathione
GST: glutathione S-transferase
GSH: glutathione
HCC: Hepatocellular Carcinoma
HE: Hematoxylin-Eosin
KEGG: Kyoto Encyclopedia of Genes and Genomes
IL-6: Interleukin-6
IL-1β: Interleukin-1β
LC-MS: liquid-chromatography-mass spectrometry
MAPK: mitogen-activated protein kinase
MRP2: multidrug resistance-associated protein 2
MRP3: multidrug resistance-associated protein 3
MRP4: multidrug resistance-associated protein 4
NRs: nuclear receptors
NTCP: Na+-dependent taurocholate cotransporting polypeptide
OATP2: organic anion transporting polypeptide 2
OCA: obeticholic acid
PBC: primary biliary cirrhosis
PFA: paraformaldehyde
PPARα: peroxisome proliferator-activated receptor alpha
PXR: Pregnane X receptor
ROS: reactive oxygen species
TBA: total bile acid
TBiL: total bilirubin
TCM: Traditional Chinese medicine
TNF-α: tumor necrosis factor alpha
3D: Three-dimensional
TLR: Toll-like receptor
UDCA: ursodeoxycholic acid.
