# Supplementary figures and images for: Da-Chai-Hu-Tang Protects From Acute Intrahepatic Cholestasis by Inhibiting Hepatic Inflammation and Bile Accumulation via Activation of PPARα
Source: Front Pharmacol. 2022 Mar 15;13:847483. doi: 10.3389/fphar.2022.847483 (PMC8965327; doi:10.3389/fphar.2022.847483)

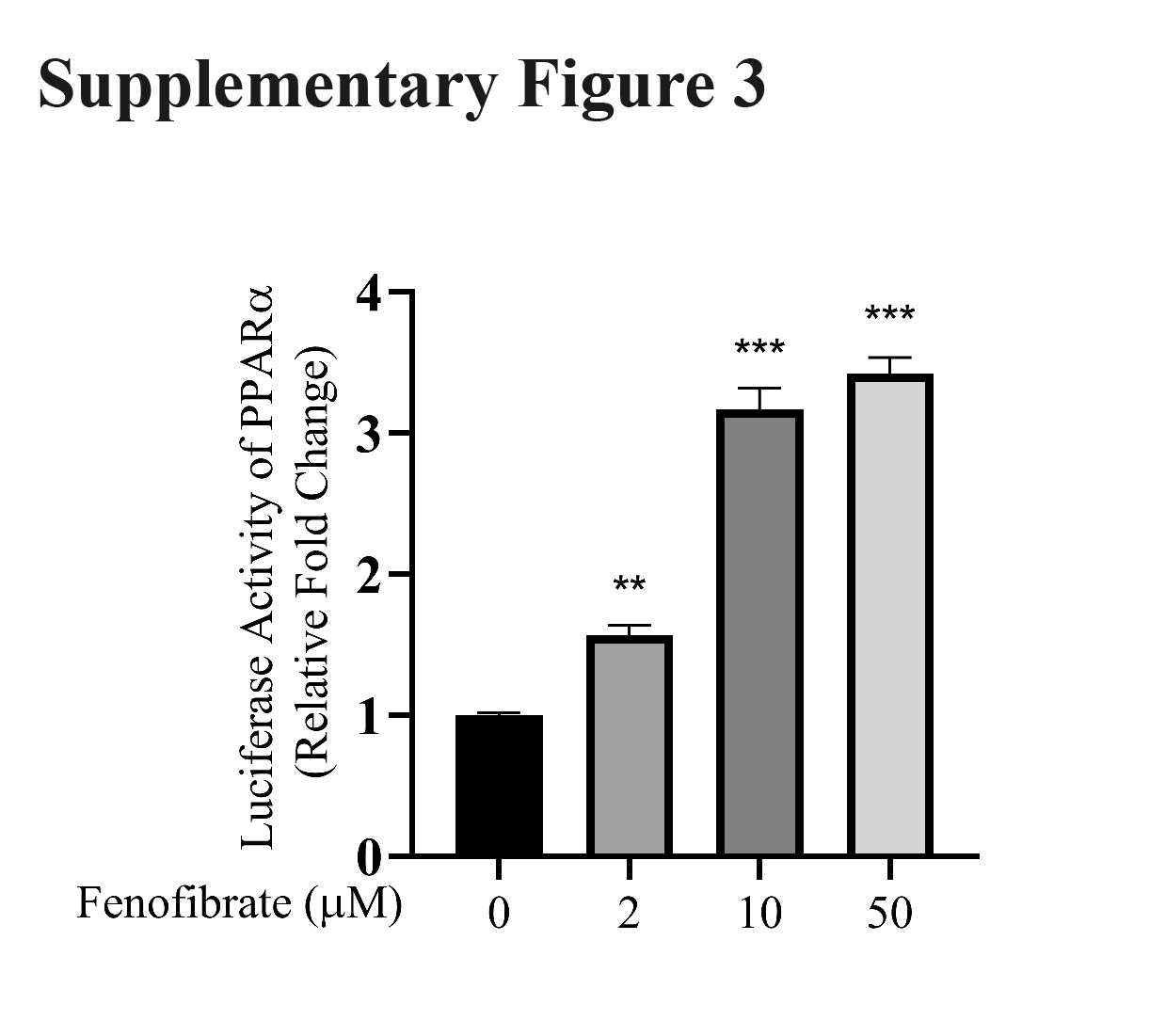

Supplement: Supplementary file 1 [file Image3.JPEG]

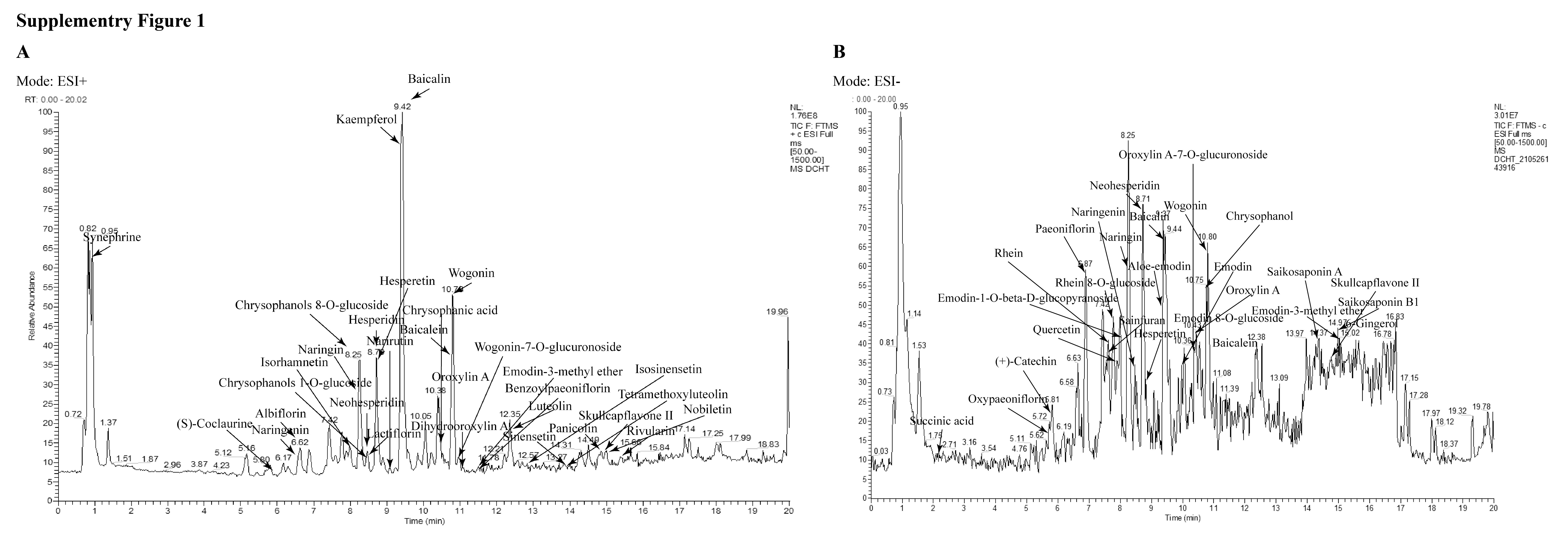

Supplement: Supplementary file 2 [file Image1.JPEG]

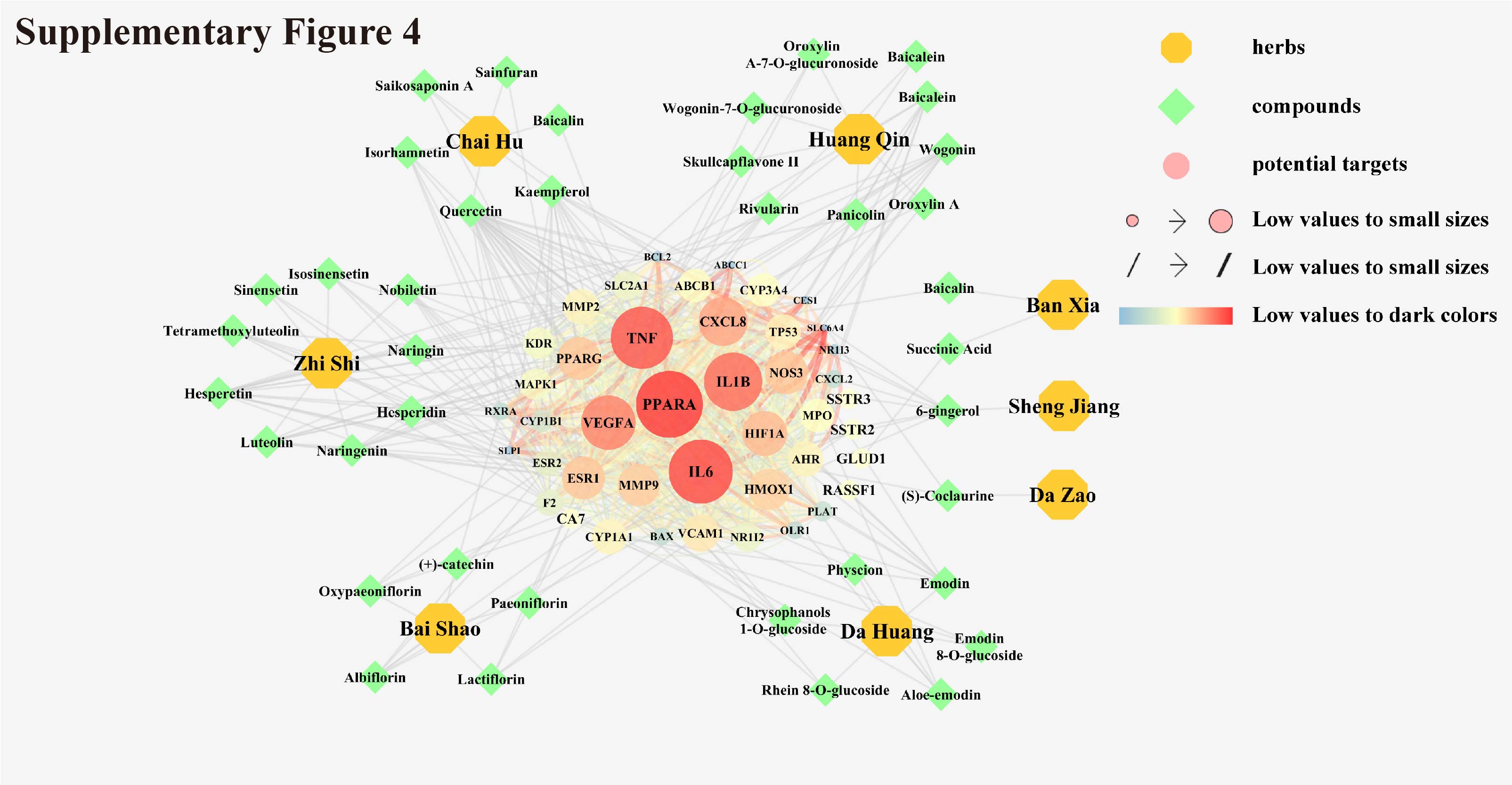

Supplement: Supplementary file 3 [file Image4.JPEG]

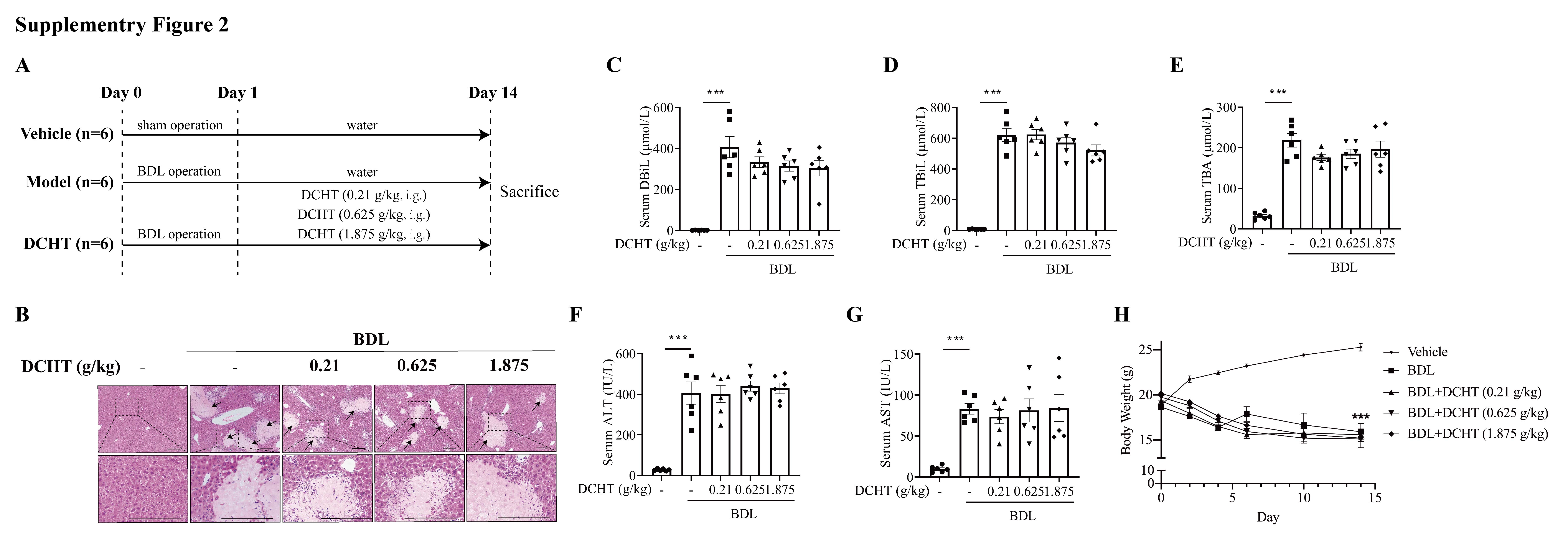

Supplement: Supplementary file 4 [file Image2.JPEG]

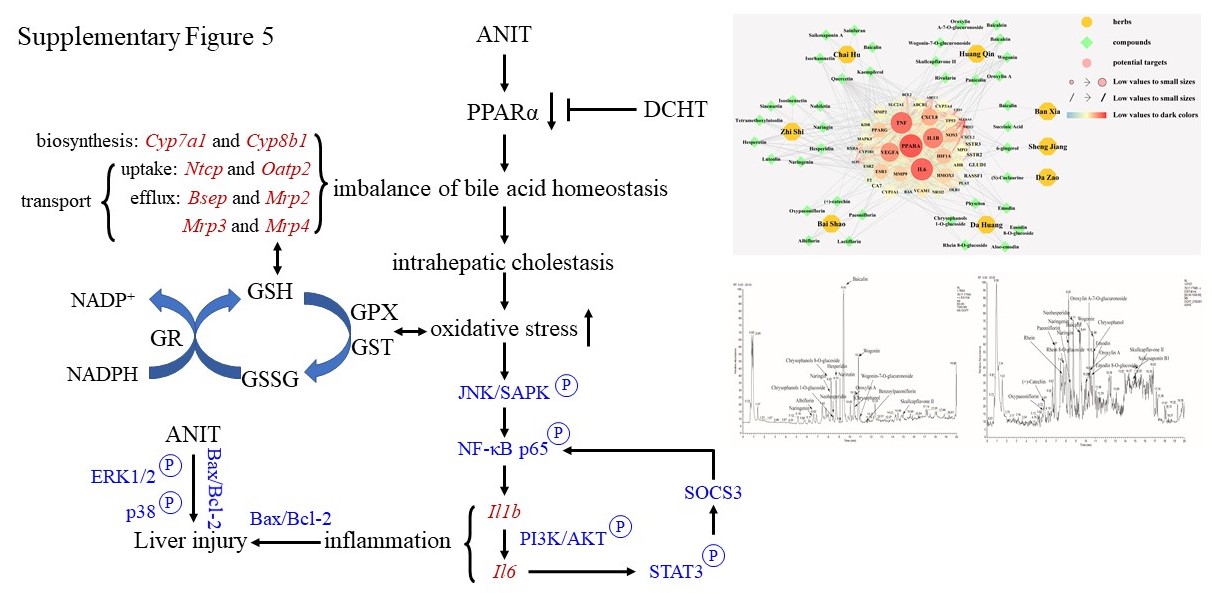

Supplement: Supplementary file 5 [file Image5.JPEG]
