# Supplementary material for: Da-Chai-Hu-Tang Protects From Acute Intrahepatic Cholestasis by Inhibiting Hepatic Inflammation and Bile Accumulation via Activation of PPARα
Source: Front Pharmacol. 2022 Mar 15;13:847483. doi: 10.3389/fphar.2022.847483 (PMC8965327; doi:10.3389/fphar.2022.847483)
Supplement: Supplementary file 6 [file DataSheet1.docx]

**Supplementary Table 1 List of reagents and antibodies used in this study.**

| **REAGENT or RESOURCE** | | **SOURCE** | **IDENTIFIER** |
| --- | --- | --- | --- |
| **Antibodies** | | | |
| phosphorylated (p-)STAT3 at Tyr705 | | Cell Signaling Technology | 9145 |
| STAT3 | | Cell Signaling Technology | 12640 |
| phosphorylated (p-)NF-ĸB p65 at Ser536 | | Cell Signaling Technology | 3033 |
| NF-ĸB p65 | | Cell Signaling Technology | 8242 |
| SOCS3 | | Cell Signaling Technology | 52113 |
| p-ERK1/2 at Thr202 and Tyr204 | | Cell Signaling Technology | 4370 |
| ERK1/2 | | Cell Signaling Technology | 4695 |
| p-p38 at Thr180 and Tyr182 | | Cell Signaling Technology | 4511 |
| p38 | | Cell Signaling Technology | 8690 |
| p-JNK/SAPK at Thr183 and Tyr185 | | Cell Signaling Technology | 4668 |
| JNK/SAPK | | Cell Signaling Technology | 9258 |
| Bcl-2 | | Cell Signaling Technology | 2876 |
| Bax | | Cell Signaling Technology | 14796 |
| p-AKT at Thr308 | | Cell Signaling Technology | 9275 |
| p-AKT at Ser473 | | Cell Signaling Technology | 9271 |
| AKT | | Cell Signaling Technology | 9272 |
| GAPDH | | Cell Signaling Technology | 2118 |
| anti-rabbit IgG, HRP-linked antibody | | Cell Signaling Technology | 7074 |
| **Chemicals** | | | |
| RIPA buffer | Cell Signaling Technology | | 9806S |
| α-naphthylisothiocyanate (ANIT) | | Sigma | N4525 |
| obeticholic acid (OCA) | | Selleck | S7660 |
| fenofibrate | | Selleck | S1794 |
| GW6471 | | Selleck | S2798 |

**Supplementary Table 2 List of primers used in this study.**

| Primer | Primer sequence |
| --- | --- |
| *Ahr* forward | 5'-AGC CGG TGC AGA AAA CAG TAA-3' |
| *Ahr* reverse | 5'-AGG CGG TCT AAC TCT GTG TTC-3' |
| *Bsep* forward | 5'-TCT GAC TCA GTG ATT CTT CGC A-3' |
| *Bsep* reverse | 5'-CCC ATA AAC ATC AGC CAG TTG T-3' |
| *Car* forward | 5'-CCC TGA CAG ACC CGG AGT TA-3' |
| *Car* reverse | 5'-GCC GAG ACT GTT GTT CCA TAA T-3' |
| *Cyp7a1* forward | 5'-GAA CCT CCT TTG GAC AAC GGG-3' |
| *Cyp7a1* reverse | 5'-GGA GTT TGT GAT GAA GTG GAC AT-3' |
| *Cyp8b1* forward | 5'-TGC AAA AGA ACT GGT GCT CAA-3' |
| *Cyp8b1* reverse | 5'-CGA ACC TTT AGG CCC TAG CAT-3' |
| *Cxcl2* forward | 5'-CCA ACC ACC AGG CTA CAG G-3' |
| *Cxcl2* reverse | 5'-GCG TCA CAC TCA AGC TCT G-3' |
| *Gadd45a* forward | 5'-CCG AAA GGA TGG ACA CGG TG-3' |
| *Gadd45a* reverse | 5'-TTA TCG GGG TCT ACG TTG AGC-3' |
| *Il1b* forward | 5'-GAA ATG CCA CCT TTT GAC AGT G-3' |
| *Il1b* reverse | 5'-TGG ATG CTC TCA TCA GGA CAG-3' |
| *Il6* forward | 5'-CCA AGA GGT GAG TGC TTC CC-3' |
| *Il6* reverse | 5'-CTG TTG TTC AGA CTC TCT CCC T-3' |
| *Mrp2* forward | 5'-ACG TTT AGT TGG TAT GAC AGC AC-3' |
| *Mrp2* reverse | 5'-TGC TTC TTG GTC AAT CCG TGT-3' |
| *Mrp3* forward | 5'-AGG AGA AGC CGC CTT TGT TC-3' |
| *Mrp3* reverse | 5'-GCA GCC GTT GTA CCA CCT T-3' |
| *Mrp4* forward | 5'-CAT CGC GGT AAC CGT CCT C-3' |
| *Mrp4* reverse | 5'-CCG CAG TTT TAC TCC GCA G-3' |
| *Ntcp* forward | 5'-CAA ACC TCA GAA GGA CCA AAC A-3' |
| *Ntcp* reverse | 5'-GTA GGA GGA TTA TTC CCG TTG TG-3' |
| *Oatp2* forward | 5'-GGG AAC ATG CTT CGT GGG ATA-3' |
| *Oatp2* reverse | 5'-GGA GTT ATG CGG ACA CTT CTC-3' |
| *Ppara* forward | 5'-AGA GCC CCA TCT GTC CTC TC-3' |
| *Ppara* reverse | 5'-ACT GGT AGT CTG CAA AAC CAA A-3' |
| *Pxr* forward | 5'-GAT GGA GGT CTT CAA ATC TGC C-3' |
| *Pxr* reverse | 5'-GGC CCT TCT GAA AAA CCC CT-3' |
| *Tnfa* forward | 5'-GAC GTG GAA CTG GCA GAA GAG-3' |
| *Tnfa* reverse | 5'-TTG GTG GTT TGT GAG TGT GAG-3' |
| *Gapdh* forward | 5'-TGA CCA CAG TCC ATG CCA TC-3' |
| *Gapdh* reverse | 5'-GAC GGA CAC ATT GGG GGT AG-3' |

**Supplementary Table 3 LC-MS based chemoprofile of DCHT.**

| **Detection Mode** | **Name** | **Formula** | **Molecular Weight** | **RT [min]** | **Area (Max.)** | **Tsumura Co.** |
| --- | --- | --- | --- | --- | --- | --- |
| ESI- | Succinic acid | C4 H6 O4 | 118.02696 | 1.955 | 26404.07677 |  |
| ESI+ | Synephrine | C9 H13 N O2 | 167.09420 | 0.945 | 228751.1601 |  |
| ESI+ | Chrysophanol | C15 H10 O4 | 254.05768 | 10.432 | 279708.8865 | Y |
| ESI- | Chrysophanol | C15 H10 O4 | 254.05813 | 10.75 | 20511.11318 | Y |
| ESI- | Aloe-emodin | C15 H10 O5 | 270.05269 | 9.393 | 448457.2151 |  |
| ESI- | Emodin | C15 H10 O5 | 270.05269 | 10.301 | 28888.20974 | Y |
| ESI- | Baicalein | C15 H10 O5 | 270.05269 | 10.669 | 32462.70421 |  |
| ESI+ | Baicalein | C15 H10 O5 | 270.05275 | 10.636 | 196214.3399 |  |
| ESI+ | Naringenin | C15 H12 O5 | 272.06746 | 6.548 | 26046.76284 | Y |
| ESI- | Naringenin | C15 H12 O5 | 272.06849 | 8.439 | 41152.6633 | Y |
| ESI- | Rhein | C15 H8 O6 | 284.03227 | 7.605 | 43767.13035 | Y |
| ESI+ | Wogonin | C16 H12 O5 | 284.06746 | 10.79 | 1588309.32 | Y |
| ESI- | Wogonin | C16 H12 O5 | 284.06828 | 10.796 | 118803.1946 | Y |
| ESI+ | Oroxylin A | C16 H12 O5 | 284.06746 | 10.393 | 565360.0085 | Y |
| ESI- | Oroxylin A | C16 H12 O5 | 284.06828 | 10.43 | 36125.57021 | Y |
| ESI+ | Emodin-3-methyl ether | C16 H12 O5 | 284.06746 | 11.767 | 43967.03089 |  |
| ESI- | Emodin-3-methyl ether | C16 H12 O5 | 284.06856 | 14.994 | 23956.56118 |  |
| ESI+ | (S)-Coclaurine | C17 H19 N O3 | 285.13640 | 5.92 | 12912.58724 |  |
| ESI+ | Luteolin | C15 H10 O6 | 286.04775 | 12.364 | 30056.17038 |  |
| ESI+ | Kaempferol | C15 H10 O6 | 286.04694 | 9.395 | 1289158.047 |  |
| ESI- | Sainfuran | C16 H14 O5 | 286.04771 | 7.686 | 14636.67943 |  |
| ESI+ | Dihydrooroxylin A | C16 H14 O5 | 286.04775 | 11.083 | 19511.13316 |  |
| ESI- | (+)-catechin | C15 H14 O6 | 290.07893 | 5.796 | 371793.7375 | Y |
| ESI- | 6-Gingerol | C17 H26 O4 | 294.18312 | 15.193 | 13594.03789 |  |
| ESI+ | Hesperetin | C16 H14 O6 | 302.07768 | 8.71 | 1819269.574 |  |
| ESI- | Hesperetin | C16 H14 O6 | 302.07884 | 8.973 | 19394.47969 |  |
| ESI- | Quercetin | C15 H10 O7 | 302.04259 | 7.861 | 149116.9905 |  |
| ESI+ | Panicolin | C17 H14 O6 | 314.31000 | 14.771 | 77084.27822 |  |
| ESI+ | Isorhamnetin | C16 H12 O7 | 316.05788 | 8.079 | 70820.84649 |  |
| ESI+ | Tetramethoxyluteolin | C19 H18 O6 | 342.11025 | 13.864 | 85820.93014 |  |
| ESI+ | Rivularin | C18 H16 O7 | 344.08949 | 15.388 | 17010.49299 |  |
| ESI+ | Sinensetin | C20 H20 O7 | 372.12068 | 13.837 | 30108.04974 |  |
| ESI+ | Isosinensetin | C20 H20 O7 | 372.12084 | 12.869 | 40770.6398 |  |
| ESI+ | Skullcapflavone II | C19 H18 O8 | 374.09950 | 14.8 | 345938.9845 | Y |
| ESI- | Skullcapflavone II | C19 H18 O8 | 374.10008 | 14.807 | 41386.20494 | Y |
| ESI+ | Nobiletin | C21 H22 O8 | 402.13110 | 14.867 | 277995.6068 |  |
| ESI+ | Chrysophanols 1-O-glucoside | C21 H20 O9 | 416.10969 | 8.461 | 220859.9835 | Y |
| ESI+ | Chrysophanols 8-O-glucoside | C21 H20 O9 | 416.10996 | 8.247 | 69843.48438 | Y |
| ESI- | Emodin 8-O-glucoside | C21 H20 O10 | 432.10533 | 10.563 | 135567.4881 | Y |
| ESI- | Emodin-1-O-beta-D-glucopyranoside | C21 H20 O10 | 432.10817 | 8.126 | 26488.61323 |  |
| ESI- | Baicalin | C21 H18 O11 | 446.08751 | 9.423 | 1137961.88 | Y |
| ESI+ | Baicalin | C21 H18 O11 | 446.08266 | 9.42 | 6961952.265 | Y |
| ESI- | Rhein 8-O-glucoside | C21 H18 O11 | 446.08894 | 7.613 | 141816.7489 | Y |
| ESI+ | Wogonin-7-O-glucuronoside | C22 H20 O11 | 460.09868 | 11.377 | 85453.8428 | Y |
| ESI- | Oroxylin A-7-O-glucuronoside | C22 H20 O11 | 460.09962 | 10.412 | 387003.7249 | Y |
| ESI+ | Lactiflorin | C23 H26 O10 | 462.14989 | 8.484 | 17523.76308 |  |
| ESI+ | Albiflorin | C23 H28 O11 | 480.16239 | 6.613 | 919605.8932 | Y |
| ESI- | Paeoniflorin | C23 H28 O11 | 480.16244 | 6.866 | 882519.1808 | Y |
| ESI- | Oxypaeoniflorin | C23 H28 O12 | 496.15742 | 5.675 | 65876.23709 | Y |
| ESI- | Naringin | C27 H32 O14 | 580.17610 | 8.247 | 2118065.151 | Y |
| ESI+ | Naringin | C27 H32 O14 | 580.17721 | 8.239 | 606753.407 | Y |
| ESI+ | Narirutin | C27 H32 O14 | 580.17919 | 9.061 | 13054.42962 | Y |
| ESI+ | Benzoylpaeoniflorin | C30 H32 O12 | 584.18895 | 11.483 | 46222.78227 | Y |
| ESI+ | Hesperidin | C28 H34 O15 | 610.18747 | 8.7 | 541956.0153 | Y |
| ESI- | Neohesperidin | C28 H34 O15 | 610.18721 | 8.711 | 1500401.871 | Y |
| ESI+ | Neohesperidin | C28 H34 O15 | 610.18765 | 8.464 | 174718.1309 | Y |
| ESI- | Saikosaponin A | C42 H68 O13 | 780.46339 | 14.311 | 171102.8226 |  |
| ESI- | Saikosaponin B1 | C42 H68 O13 | 780.46340 | 14.974 | 45167.65115 | Y |

**Supplementary Table** **4 List of DCHT potential targets.**

| Term | Number | Targets |
| --- | --- | --- |
| Overlap targets of DCHT and intrahepatic cholestasis and extrahepatic cholestasis | 20 | ABCB1, ABCC1, BAX, BCL2, CXCL8, F2, HIF1A, IL6, KDR, MAPK1, MMP2, MMP9, MPO, SLC2A1, SLPI, SSTR2, SSTR3, TNF, TP53, VEGFA |
| Exclusively targets of DCHT and intrahepatic cholestasis | 22 | AHR, CXCL2, ESR1, ESR2, NR1I2, NR1I3, PPARA, CA7, CES1, CYP1A1, CYP1B1, CYP3A4, GLUD1, HMOX1, IL1B, OLR1, PLAT, NOS3, RXRA, SLC6A4, VCAM1, PPARG |
| Exclusively targets of DCHT and extrahepatic cholestasis | 69 | ACTA2, AKT1, AURKA, BCL2L1, BIRC5, CA2, CASP3, CASP7, CASP8, CASP9, CAV1, CCNA2, CCND1, CDK2, CDKN1A, CHUK, CLDN4, CRP, CYCS, E2F1, E2F2, EGFR, ERBB2, ERBB3, FGF1, FGF2, FGFR1, FLT4, FN1, FOS, GAPDH, GSK3B, HK2, HPSE, HRAS, HSP90AA1, HSPA5, HSPB1, IKBKB, JUN, LGALS3, MAP2K1, MAPK14, MAPK3, MAPK8, MCL1, MDM2, MET, MMP7, MYC, NFE2L2, NFKBIA, NOS2, PARP1, PCNA, PDP1, PLAU, PRKCA, PRSS1, PTGS2, RAF1, RB1, RELA, SLC29A1, SRC, STAT1, TIMP1, TOP1, XDH |

**Supplementary Table 5 List of exclusively targets of DCHT and intrahepatic cholestasis from two database.**

| Term | Number | Targets |
| --- | --- | --- |
| Overlap exclusively targets of DCHT and intrahepatic cholestasis from two database | 7 | AHR, CXCL2, ESR1, ESR2, NR1I2, NR1I3, PPARA |
| Exclusively targets of DCHT and intrahepatic cholestasis from GeneCards database | 14 | CA7, CES1, CYP1A1, CYP1B1, CYP3A4, GLUD1, HMOX1, IL1B, OLR1, PLAT, NOS3, RXRA, SLC6A4, VCAM1 |
| Exclusively targets of DCHT and intrahepatic cholestasis from DisGeNET database | 1 | PPARG |

**Supplementary Figure 1 LC-MS based chemoprofile of a methanol solution of DCHT.** The detection modes are ESI+ (A) and ESI- (B), respectively.

**Supplementary Figure 2** **DCHT has no effect on BDL-induced cholestatic liver injury *in vivo*.**

**(A)** Scheme of the experimental design. **(B)** H&E-stained liver sections. Scale bar = 200 μm. Arrows indicate the area of severe liver necrosis and hyperplastic bile cytoderm. **(C-G)** Serum levels of DBiL **(C)**, TBiL **(D)** and TBA **(E)**, ALT **(F)** and AST **(G)**. **(H)** Body weights of all animals were recorded daily. Data are shown as means ± S.E.M.; ^***^*P* < 0.001 as compared with the vehicle group, *n* = 6.

**Supplementary Figure 3** Luciferase activity of PPARα was determined after treatment with PPARα agonist fenofibrate (0-50 μM). Data are shown as means ± S.E.M.; ^**^*P* < 0.01, ^***^*P*<0.001 compared with control, *n* = 3.

**Supplementary Figure 4** Schematic illustration of the Component-Target network. The yellow octagons represent herbs contained in DCHT, while the green diamonds denote chemical compounds from DCHT, and the pink circles refer to 42 potential targets of DCHT.

**Supplementary Figure 5** Signaling pathway by which DCHT protects from acute intrahepatic cholestasis with liver injury in response to ANIT via activation of PPARα.
